# Considerations Regarding the Cytotoxicity of Certain Classes of Fungal Polyketides—Potential Raw Materials for Skincare Products for Healthy and Diseased Skin

**DOI:** 10.3390/pharmaceutics17060759

**Published:** 2025-06-09

**Authors:** Daniela Albisoru, Nicoleta Radu, Raluca Senin, Mihai Dan Caramihai, Mihaela Begea, Oksana Mulesa, Viviana Roman, Marinela Bostan

**Affiliations:** 1 Faculty of Biotechnology, University of Agronomic Sciences and Veterinary Medicine of Bucharest, 011464 Bucharest, Romania; 2 Department of Biotechnology, National Institute of Chemistry and Petrochemistry R&D of Bucharest, 060021 Bucharest, Romania; 3 Faculty of Automatic Control Computer Sciences, National University of Sciences and Technology Politehnica Bucharest, 060042 Bucharest, Romania; m.caramihai@yahoo.com; 4 Faculty of Biotechnical Systems Engineering, National University of Sciences and Technology Politehnica Bucharest, 060042 Bucharest, Romania; ela_begea@yahoo.com; 5 Faculty of Humanities and Natural Sciences, Prešov University, Námestie legionárov 3, 080 01 Prešov, Slovakia; mulesa.oksana@gmail.com; 6 Center of Immunology, Institute of Virology, Stefan S. Nicolau, 030304 Bucharest, Romania; rviviana30@yahoo.com (V.R.); marinela.bostan@yahoo.com (M.B.)

**Keywords:** fungal *polyketides*, cytotoxicity evaluation, in silico, in vitro

## Abstract

**Background**: This study investigates the cytotoxicity of microbial polyketides biosynthesized by *Monascus* species through both in silico and in vitro approaches. **Methods**: Six main know *Monascus*-derived polyketides were analysed in silico an an vitro. **Results**: In silico tests reveal that the main derived compounds exhibit lipophilic properties, indicating their potential suitability as active ingredients in dermato-cosmetic formulations. In silico tests revealed significant flexibility and high degrees of unsaturation for some *Monascus*-derived polyketides, suggesting a broad interaction potential and a propensity for chemical instability. In silico permeability tests indicated low epidermal penetration. Cytotoxicity assays conducted in vitro on a HaCaT cell line revealed varying levels of cytotoxicity among the three classes of fungal polyketides. Yellow polyketides derived from *Monascus purpureus* and *Monascus ruber* exhibited moderate cytotoxicity, while orange polyketides derived from the same strains showed low cytotoxicity. Red, orange, and yellow polyketides derived from a high-productive *Monascus* sp. genus showed low or negligible cytotoxicity. After 48 h of exposure, the cytotoxic profiles of all *Monascus* polyketides remained relatively stable. The IC_50_ values obtained through linear or nonlinear models supplied by EXCEL MS Office or for the Systat programme indicated moderate-to-low cytotoxicity for polyketides derived from *Monascus ruber* and *Monascus purpureus*. The bioproducts derived from high-productive *Monascus* sp. exhibited weak or negligible cytotoxicity. **Conclusions**: The results obtained suggest that the *Monascus*-derived polyketides possess promising properties for therapeutic and cosmetic applications, but their chemical stability must be considered in the case of dermatological formulations.

## 1. Introduction

The coloured polyketides produced by fungal microorganisms of the *Monascus* genus are widely used in Asia within the food industry to colour products such as yoghurt, cheese, sweets, and ice cream [1,2,3,4]. Studies conducted to date have shown that polyketides biosynthesized by *Monascus* species exhibit antioxidant [5,6,7,8], antiproliferative, antibacterial, and antifungal activities [9,10,11,12,13], making them interesting for potential dermato-cosmetic applications. These bioproducts are often used in Asia as dietary supplements with hypocholesterolaemic effects [1,14], although further studies are needed to assess their safety in patients with health conditions, due to the presence of mycotoxins, the non-standardised content of monacolins [15,16,17,18], and the presence of polyketides [13]. An interesting approach relates to the use of these compounds in dermato-cosmetic formulations. Over time, microbial polyketides derived from *Monascus* species have been the subject of various patents and scientific publications. For example, a Korean patent [19] describes cosmetic biopreparations with antioxidant and anti-ageing effects containing oils fermented with *Monascus* sp. The raw material is obtained by fermenting plant seeds (jojoba, *Camellia*, green tea, macadamia, and sunflower seeds) or dried fruits (dried olives) with *Monascus* species. After fermentation, the fermented seeds or fruits are steam-sterilised and then pressed to obtain oil. The resulting oils are used as raw materials in the development of dermato-cosmetic products for skincare [19].

**Figure 1:**
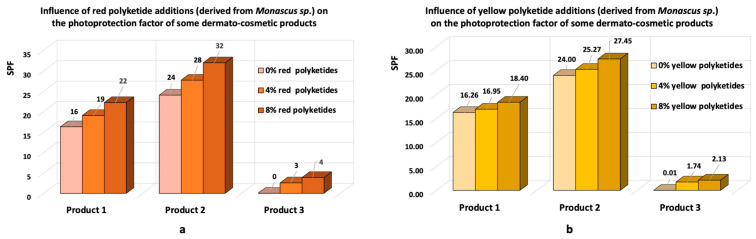
The effects of *Monascus*-derived polyketides in dermato-cosmetic formulations: (**a**) the presence of red polyketides derived from *Monascus* sp. enhances the sunscreen protection factor in three types of skincare products (adaptation after the data reported by Koli et al. [21]); (**b**) the presence of yellow polyketides derived from *Monascus* sp. enhances the sunscreen protection of three types of skincare products (adaptation after the data reported by Koli et al. [21]).

Another patent, granted to L’Oréal Paris, France [20], describes dermato-cosmetic bioproducts with tanning effects that contain polyketides obtained through solid-state biosynthesis (biosynthesis on solid substrate), using various *Monascus* species. The polyketides described in this patent are obtained by selective extraction with organic solvents and then are spray-dried in the presence of maltodextrin (4 parts Monascus to 96 parts maltodextrin). The resulting bioproducts are used as a raw material in the formulation of various dermato-cosmetic products with tanning properties. Koli and collaborators [21], in the studies carried out with red and yellow polyketides biosynthesised by *Monascus* sp., demonstrated that when these compounds are added to dermato-cosmetic formulations at concentrations ranging from 4% to 8%, they enhance their photoprotective properties (Figure 1a,b).

**Figure 2:**
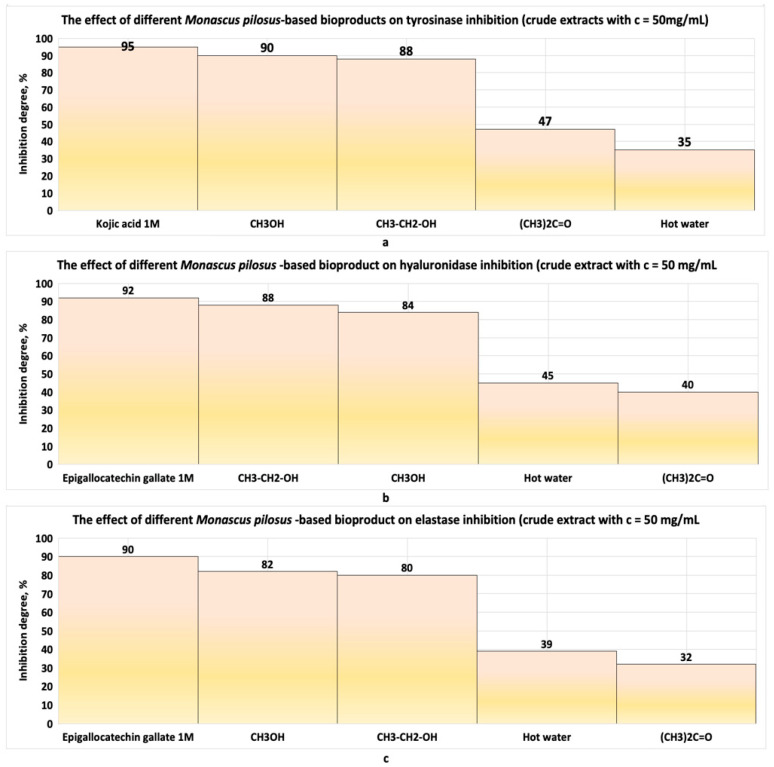
The anti-ageing effect of different derived *Monascus pilosus* bioproducts: (**a**) anti-tirozinase effect; (**b**) anti-hyaluronidase effect; (**c**) anti-elastase effect. Adaptation after the data reported by Jin and Pyo [22].

**Figure 3:**
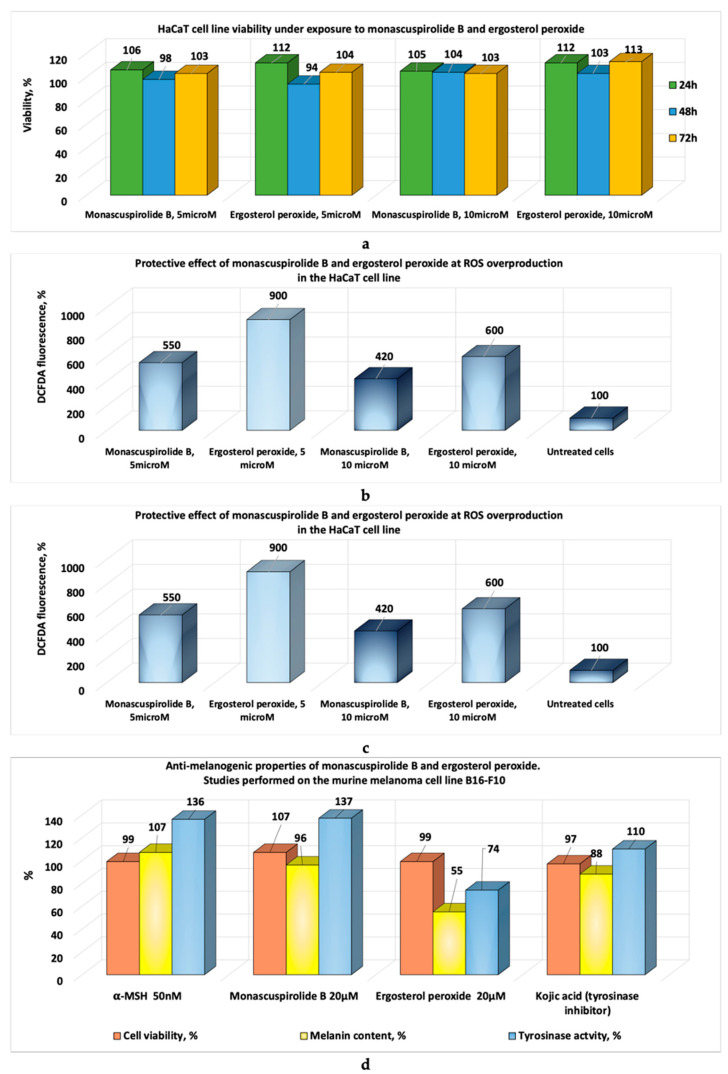
The potential dermato-cosmetic applications of the compounds Monascuspirolide B and Ergosterol peroxide: (**a**) the influence on HaCaT proliferation; (**b**) the protective effect against UV-B radiation on HaCaT; (**c**) protective effect against ROS overproduction in HaCaT; (**d**) antimelanogenic properties (adaptation after Wu et al. [23]).

**Figure 4:**
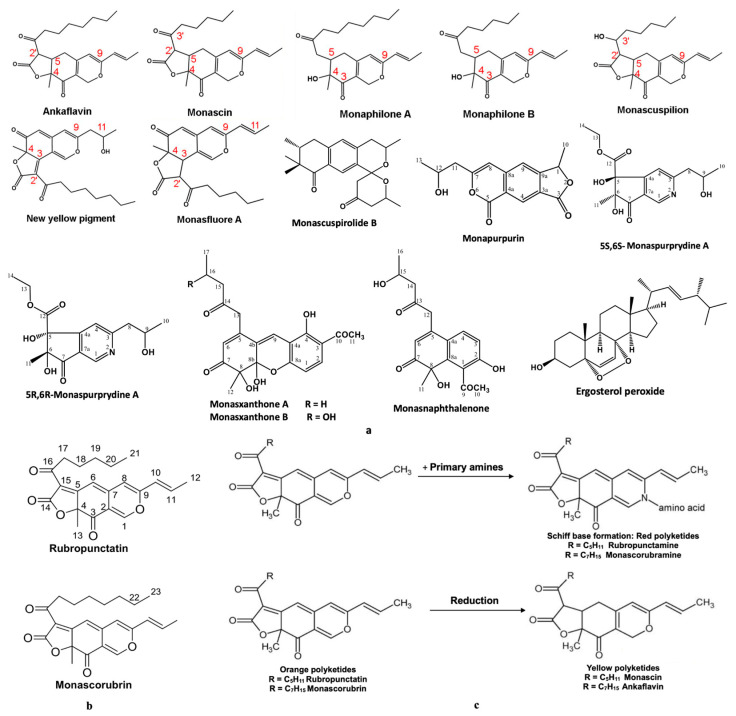
The main coloured polyketides biosynthesised by species from the *Monascus* genus, in connection with photoprotective and anti-melanogenic activities: (**a**) yellow and white polyketides (i.e., Ergosterol peroxide); (**b**) orange polyketides (adaptation after Wu et al. [23]). Monascuspirolide B, based on its structure, may have a yellow–orange colour; (**c**) yellow and red polyketides’ biosynthesis from orange polyketides; adaptation after de Oliveira et al. [24].

Jin and Pyo, in the studies made with soybean extracts fermented with *Monascus*, reported that these bioproducts inhibit elastase and collagenase enzymes involved in collagen and elastin degradation in the skin (Figure 2a–c). These properties recommend *Monascus*-derived polyketides as potential raw materials for dermato-cosmetic bioproducts with anti-ageing effects [22]. In vitro tests performed by Wu and collaborators [23] on a murine melanoma cell line and a human keratinocyte cell line type, HaCaT, with two compounds (Monascuspirolide and Ergosterol peroxide) isolated from an alcoholic extract of *Monascus*-fermented rice showed that these compounds: (1) do not affect keratinocyte viability (Figure 3a); (2) protect keratinocytes from UV-B radiation (Figure 3b); (3) reduce the reactive oxygen species (Figure 3c); and (4) exhibit anti-melanogenic properties (Figure 3d) [22]. Additionally, soybean extracts fermented with Monascus showed significant free radical-scavenging activity (DPPH and ABTS tests), protecting HaCat cells from oxidative stress. These extracts inhibit enzymes involved in skin ageing, such as elastase and collagenase, a property that supports their use as raw materials in anti-ageing dermato-cosmetic formulations [23]. The coloured polyketides of interest for such formulations include monascorubramine, monascorubrin, rubropunctamine, rubropunctatin, monascin, and ankaflavin, though *Monascus* species produce many more coloured polyketides (Figure 4a–c). Notably, orange polyketides (Figure 4b) are important because they are biosynthesized first. The orange polyketides (two structures presented in the Figure 4b) may be reduced to yellow polyketides [22,24] with chemical structures presented in the Figure 4a, or can be turned into red–purple polyketides in the presence of the primary amines (chemical reactions presented in the Figure 4c) [24].

The recent in silico studies conducted by Albișoru and colleagues [13] have shown that six of the polyketides synthesised by *Monascus* sp. possess antimicrobial properties but exhibit hepatic and neurological toxicity, making bioproducts containing them more suitable for topical formulations [13]. In this context, the studies aimed to achieve the following objectives:

1. In silico prediction of the properties of six of the most well-known polyketides biosynthesized by Monascus, and an evaluation of their dermal and oral absorption capacity;
2. In vitro evaluation of the effect of bioproducts containing yellow and orange polyketides on the viability of standardised HaCaT cell lines;
3. Evaluation of the IC50 value (the concentration at which a substance exerts half of its maximum effect) based on in vitro data, using linear and nonlinear models supplied by the SYSTAT version 13.2, (Inpixon, Palo Alto, CA, USA) or ATT Bioquest programme version 1.0.0, (Pleasanton, CA, USA);
4. Evaluation of the cytotoxicity of three types of bioproducts enriched with coloured polyketides, derived from *Monascus purpureus*, *Monascus ruber*, and a high-productive *Monascus* sp.

## 2. Materials and Methods

### 2.1. Bioproducts Obtaining

The polyketides used in this study were biosynthesised by three *Monascus* species, as follows: *Monascus purpureus* DSM 1379 (MP), *Monascus ruber* MUCL 28,962 (MR), and a high-yielding *Monascus* species (MM), the latter having been received as a gift from Prof. Octavian Duliu of the Institute of Atomic Physics, Măgurele, Romania [13].

Three classes of coloured polyketides were obtained according to the methodology reported by Albisoru and collaborators [13], via solid-state biosynthesis, from each *Monascus* strain and *Oryza sativa* as a solid substrate. These three classes, including six well-known polyketides (namely monascorubrin, monascorubramine, rubropunctatin, rubropunctamine, monascin, and ankaflavin) were produced by each strain, their presence confirmed by LC-MS TOF in our previous work [13]. The difference between each bioproduct lies in the fact that the crude extract obtained from solid-state biosynthesis with MM contains a higher quantity of coloured polyketides compared with the bioproducts resulting from biosynthesis with MR or MP strains. The three classes of coloured *Monascus* polyketides (red, orange, and yellow polyketides) were obtained from the bioproduct resulting from solid-state biosynthesis, by extraction with selective solvents, using the methodology presented in [13]. In this study, we used the crude polyketides dissolved in an equal volume of dimethyl sulphoxide (DMSO) [13].

### 2.2. In Silico Studies

The degree of toxicity was assessed taking into account the value of IC50 and levels indicated by NIEHS-US and other reports [25,26,27,28,29,30,31] from the tests performed in vitro on cell lines. The radar maps and the boiled egg-type diagram regarding the bioavailability of the six main know *Monascus* polyketides previously identified [13] in the analysed bioproducts were generated using the Swiss ADME programme. The data regarding the skin permeability for the six *Monascus*-derived polyketides were obtained from pharmacokinetic parameters predicted in silico by the same Swiss ADME programme.

### 2.3. In Vitro Studies

Cytotoxicity tests were conducted on a standardized HaCaT cell line, obtained from Cell Line Service GmbH (cat. no. 330493, Eppenheim, Germany), using the methodology reported by Bostan et al. [25]. The human immortalized keratinocyte cell line, HaCaT, was provided by Cell Line Service GmbH (Cat no. 330493, Eppelheim, Germany) and was grown and maintained in Dulbecco’s modified Eagle’s medium (DMEM) with 10% foetal bovine serum and 1% antibiotics (10,000 μg/mL streptomycin and 10,000 units/mL penicillin) at 30 °C with 5% CO_2_. Cells were serially passaged at 70–80% confluence. When performing treatments, HaCaT cells were grown to 70–80% confluence after 24 h. Cytotoxicity data for red polyketides are available in our previously published paper (Albisoru et al. 2025, accepted, in press [32]) and for this reason are not presented in detail in this manuscript. All measurements were performed in triplicate, and the results are expressed as average values with corresponding standard deviations (STDEVs). The exclusion of the positive control at this stage was motivated by the initial screening nature of the test, in which the validation of the method was achieved through the predictable behaviour of the negative control and standard cell culture parameters. In subsequent stages, validated positive controls will be included to calibrate the biological response. In this stage, all results were compared with a negative control (untreated cells). The tests performed with the solvent used (DMSO), at the corresponding dilutions, did not show any influence on the HaCaT cell line.

### 2.4. IC50 Evaluation

The data obtained from the in vitro tests were used to evaluate the IC50 value, employing linear methods provided by Excel Office 2021 and nonlinear models supplied by the SYSTAT version 13.2, (Inpixon, Palo Alto, CA, USA) and/or ATT BioQuest model version 1.0.0 [27] (AAT Bioquest, Inc. (Pleasanton, CA, USA)). The initial concentrations of the crude polyketides in DMSO used in this study were as follows: *Monascus purpureus* (MP)-derived yellow polyketides: 125.33 mg/L; *Monascus ruber* (MR)-derived yellow polyketides: 32.59 mgL mg/L; *Monascus* high-productive (MM)-derived yellow polyketides: 695.25 mg/L; MP-derived orange polyketides: 1600 mg/L; MR-derived orange polyketides: 400 mgL mg/L; and MM-derived orange polyketides: 4800 mg/L [13]. All bioproducts were tested using the same volumetric concentration range (0–100 µL/mL). The end concentration of ethanol in cell culture media was less than 0.1%. The values of IC50 obtained are reported in µg/mL, taking into account the crude bioproduct concentration of each solution tested. The value of IC50 was assessed with linear models supplied by EXCEL MS Office, nonlinear models supplied by SYSTAT, and a nonlinear model of AAT Bioquest. Mathematical predictions generated by the SYSTAT programme were selected based on the value of the R^2^ coefficient. Only values of R^2^ > 0.81 were considered to indicate a good correlation between the mathematical model and the experimental data. In the nonlinear model used by ATT BioQuest, three cytotoxicity values obtained from in vitro tests on the HaCaT cell line were used for each concentration. Based on these values, the ATT BioQuest model generated the IC50 values, after function (1), presented below, without generating any additional statistical parameters.

(1) $$Y=Min+\frac{Max-Min}{1+{\left(\frac{X}{IC50}\right)}^{Hill\;coefficient}}$$

Microsoft Excel was used to calculate the standard deviations of these average values, which were also presented as error bars in the graphical representations of the original data obtained in vitro.

## 3. Results

### 3.1. In Silico Studies

**Figure 5:**
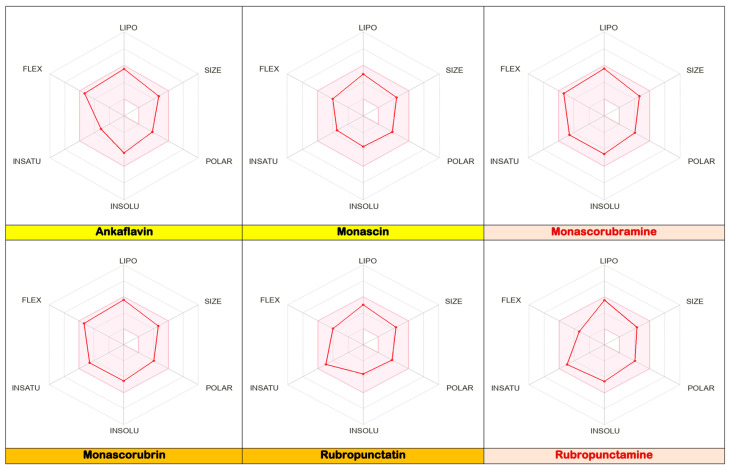
Bioavailability radar charts for the most well-known *Monascus* sp.-derived polyketides. The coloured zone indicates the physicochemical space suitable for good oral bioavailability. Diagrams generated in silico by the Swiss ADME programme.

**Figure 6:**
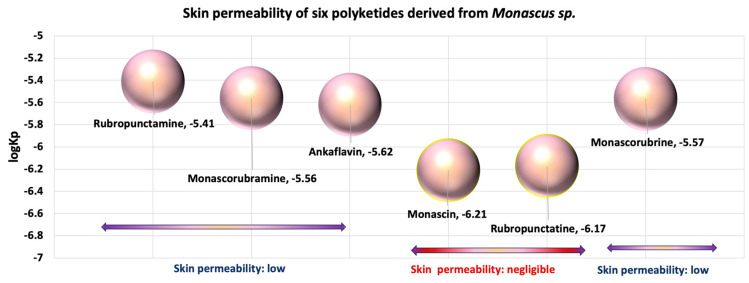
Representation of the logKp value for the main *Monascus*-derived polyketides. Values generated in silico by the Swiss ADME programme. Adaptation after Albisoru et al. [13].

**Figure 7:**
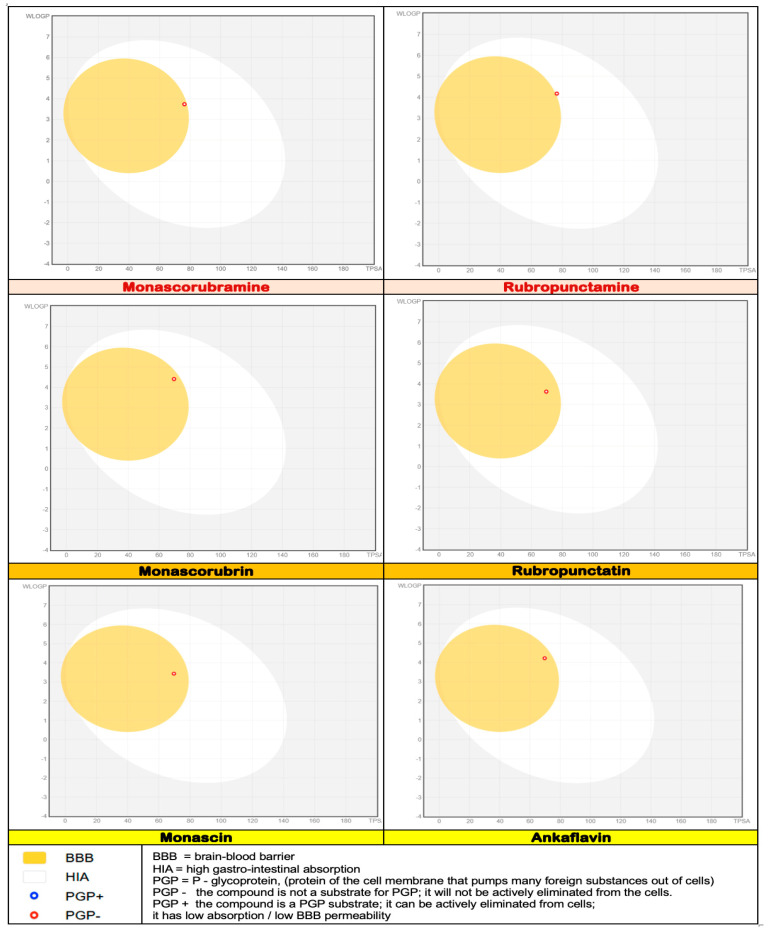
Boiled egg diagram for six *Monascus*-derived compounds.

After analysing the data obtained in silico (Figure 5, Figure 6 and Figure 7) for six of the most well-known microbial polyketides biosynthesized by *Monascus* species [13], the following observations can be made:

1. The six analysed molecules exhibit a pronounced lipophilic character (Figure 5); the partition coefficients between octanol and water (WlogP_o/w_) for the six derived *Monascus* polyketides are in the range of (3 ÷ 4) [13]. As a result, these polyketides have a greater affinity for the lipid phase than the aqueous phase, which presents them as potential excipients or valuable active ingredients for dermato-cosmetic formulations, a fact also confirmed by the other scientists [19,20,21,22,23];
2. Ankaflavin, monascorubramine, and monascorubrin are molecules with high molecular flexibility (Figure 5), which gives them the ability to interact with a wide range of biological targets. These data are supported by their antioxidant and antitumor effects [4,6,10,12,14];
3. The polyketides rubropunctatin, rubropunctamine, monascorubramine, and monascorubrin are molecules with a high degree of unsaturation (Figure 5), which provides them with a high level of chemical instability. This characteristic may limit their use in topical pharmaceutical formulations due to the possibility of rapid degradation or undesirable interactions with other compounds in the formulation.
4. Given that in silico tests indicated a potential for increased cytotoxicity, the analysis of the permeability coefficients (logKp), determined in silico by Albișoru and collaborators [13], shows that they fall within the range of [−5 ÷ −7] (Figure 6). These values suggest the low permeation of these molecules through skin layers [33,34]. In general, the six polyketides have a low capacity to pass through skin layers, with monascin and rubropunctatin exhibiting negligible permeation (logKp < −6); The “boiled egg” diagrams [35], generated in silico (Figure 7), indicate that the six analysed polyketides are not substrates for P-glycoprotein (PGP), suggesting that they are not easily eliminated from cells. The analysed molecules are easily absorbed from the gastrointestinal tract, with most of them having the ability to cross the blood–brain barrier, except for rubropunctamine.

### 3.2. In Vitro Studies

**Figure 8:**
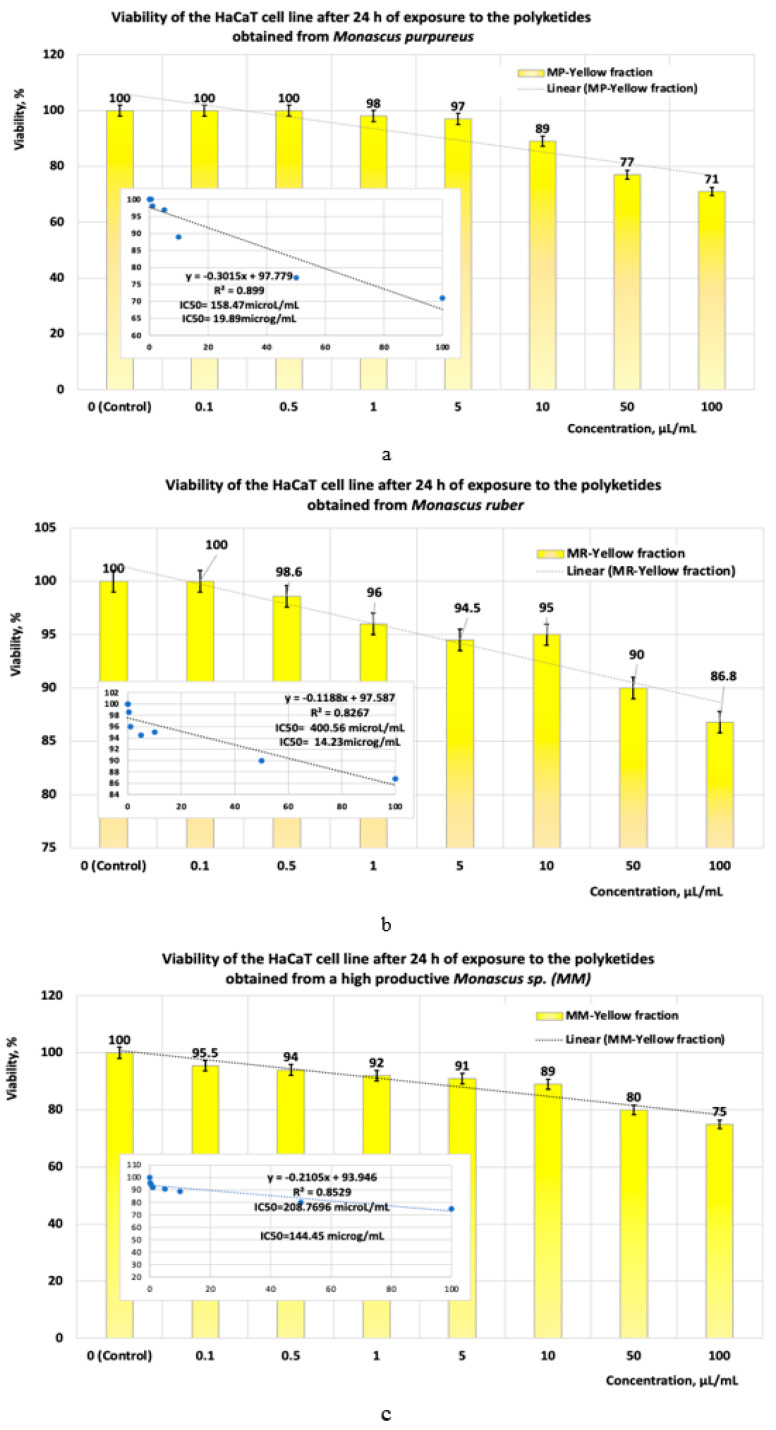
The influence of yellow *Monascus*-derived polyketides on HaCaT cell line viability after an exposure time of 24 h. Evaluation of IC50 after 24 h of exposure, with linear models for yellow polyketides derived from (**a**) *Monascus purpureus*; (**b**) *Monascus ruber*; (**c**) high-productive *Monascus* sp.

**Figure 9:**
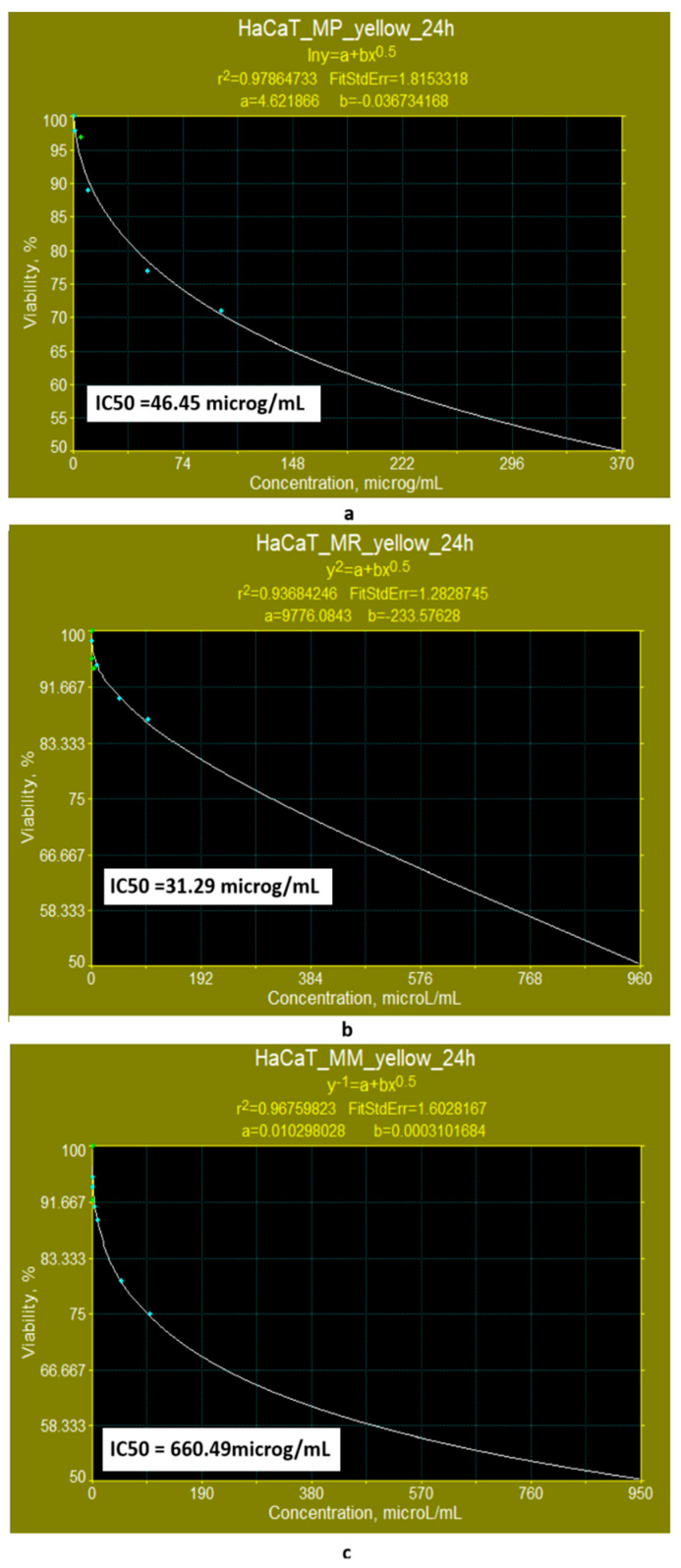
Evaluation of the IC50 value after 24 h of exposure, with nonlinear models supplied by the SYSTAT programme for yellow polyketides derived from (**a**) *Monascus purpureus*; (**b**) *Monascus ruber*; (**c**) high-productive *Monascus* sp.

**Figure 10:**
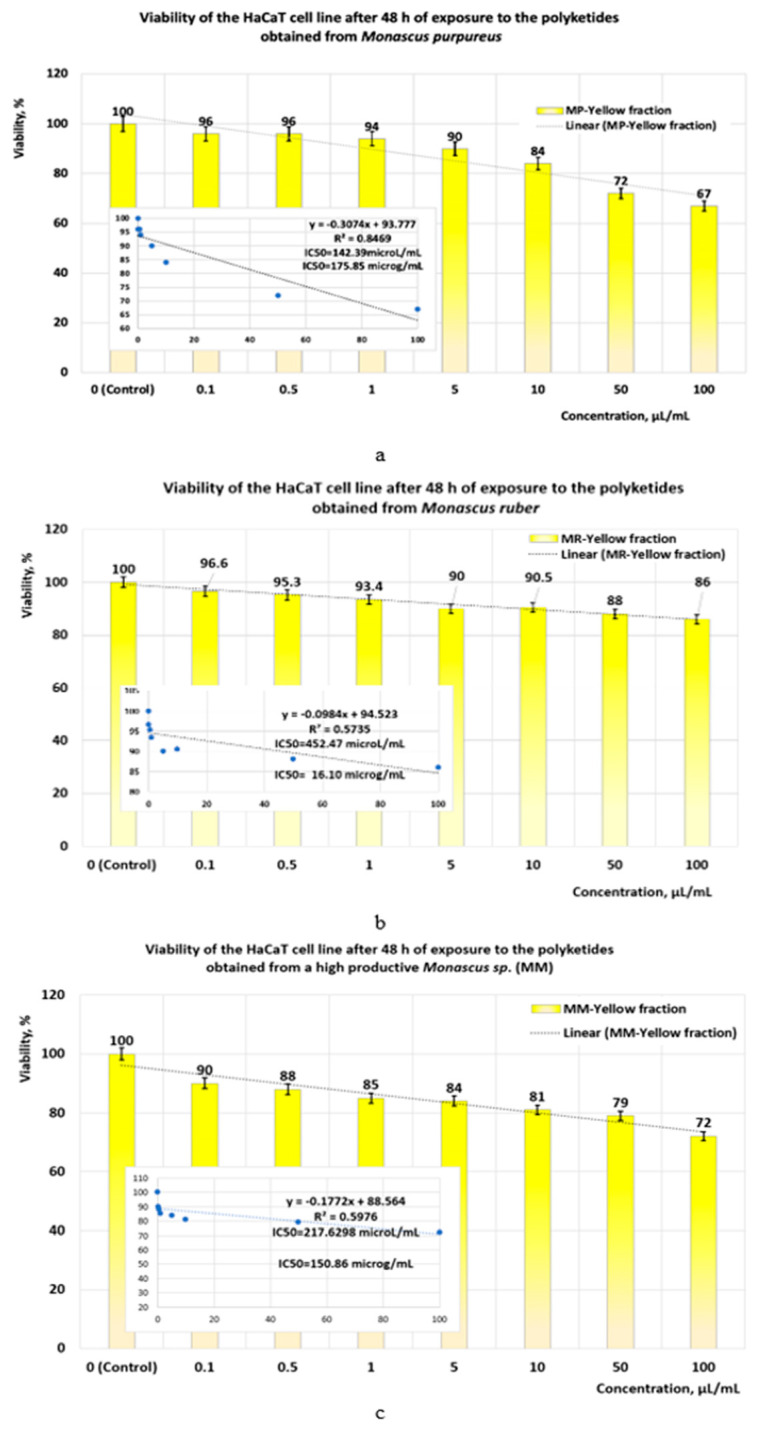
The influence of yellow *Monascus*-derived polyketides on HaCaT cell line viability after an exposure time of 48 h. Evaluation of IC50 after 48 h of exposure, with linear models for yellow polyketides derived from (**a**) *Monascus purpureus*; (**b**) *Monascus ruber*; (**c**) high-productive *Monascus* sp.

**Figure 11:**
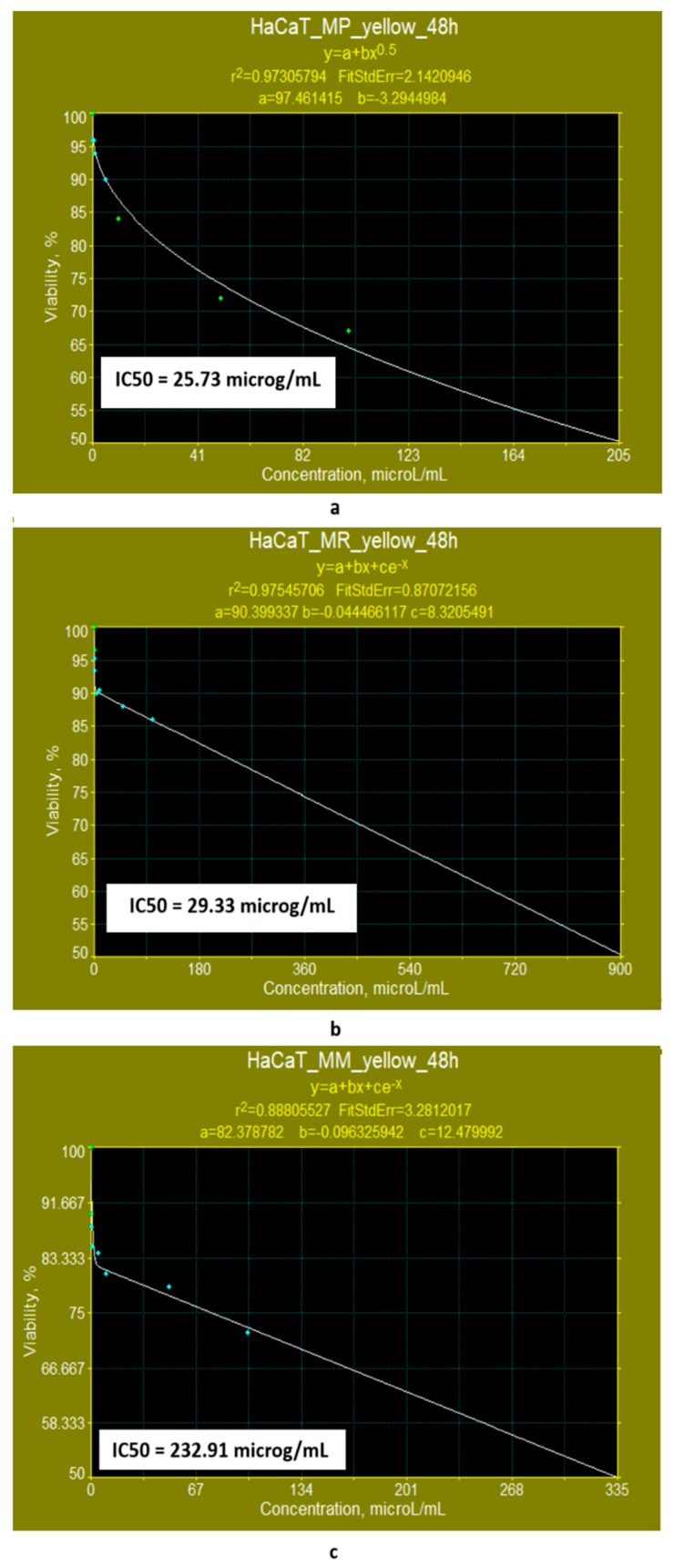
Evaluation of the IC50 value after 48 h of exposure, with nonlinear models supplied by the SYSTAT programme for yellow polyketides derived from (**a**) *Monascus purpureus*; (**b**) *Monascus ruber*; (**c**) high-productive *Monascus* sp.

The cytotoxicity tests performed in vitro on HaCaT cell lines showed the following:

(a) When the cell line is exposed for 24 h to yellow polyketides biosynthesised by the three *Monascus* species, the viability measured is greater than 71% in all three cases (Figure 8a–c). The use of linear models for evaluating the IC50 parameter shows that the yellow polyketides isolated from biopreparations derived from *Monascus purpureus* and *Monascus ruber* are cytotoxic (IC50 < 20 μg/mL). The yellow polyketides derived from high-productive *Monascus* sp. exhibit weak cytotoxicity (IC50 = 144.45 μg/mL). The evaluation of the IC50 parameter with nonlinear models provided by Microsoft SYSTAT version 13.2 (Figure 9a–c) shows that the yellow polyketides derived from *Monascus purpureus* and *Monascus ruber* (Figure 9a,b) exhibit moderate cytotoxicity (IC50 = 46.45 μg/mL and 31.29 μg/mL, respectively). In contrast, the IC50 value obtained for the yellow polyketides derived from highly productive *Monascus* sp. indicates weak cytotoxicity (IC50 = 660.49 μg/mL) (Figure 9c).

(b) If the exposure time is increased to 48 h, the linear models for evaluating the IC50 parameter (Figure 10a–c) show that the yellow polyketides derived from *Monascus ruber* retain their cytotoxic character (IC50 = 16.10 μg/mL) (Figure 10b). However, the yellow polyketides derived from *Monascus purpureus* and highly productive *Monascus* sp. exhibit weak cytotoxicity (IC50 = 175.85 μg/mL and IC50 = 150.86 μg/mL, respectively) (Figure 10a,c). Math modelling with the Systat programme confirms the in vitro results (Figure 11a–c), indicating that the yellow polyketides derived from *Monascus purpureus* and *Monascus ruber* exhibit moderate cytotoxicity (IC50 = 25.73 μg/mL and 29.33 μg/mL, respectively) (Figure 10a,b). The data obtained for yellow polyketides derived from highly productive *Monascus* sp. show that they have low cytotoxicity (IC50 = 232.91 μg/mL) (Figure 11c).

**Figure 12:**
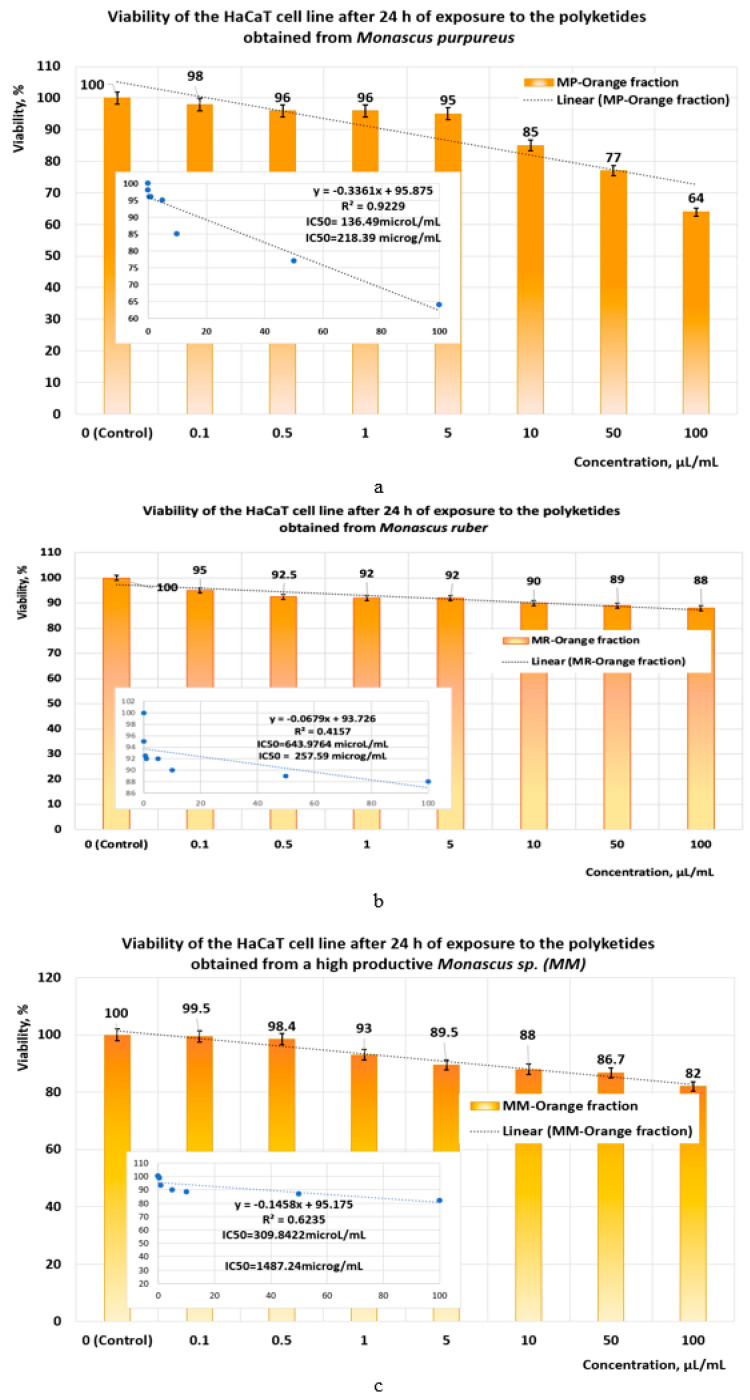
The influence of orange *Monascus*-derived polyketides on HaCaT cell line viability after an exposure time of 24 h. Evaluation of IC50 after 24 h of exposure, with linear models for orange polyketides derived from (**a**) *Monascus purpureus*; (**b**) *Monascus ruber*; (**c**) high-productive *Monascus* sp.

Following the exposure of the HaCaT cell line to orange polyketides for 24 h, the IC50 values obtained with linear models (Figure 12a–c) show that the polyketides derived from *Monascus purpureus* and *Monascus ruber* have low cytotoxicity (IC50 = 218.39 μg/mL and IC50 = 257.59 μg/mL, respectively) (Figure 12a). In the case of orange polyketides derived from highly productive *Monascus* sp., the IC50 value (IC50 = 1487.24 μg/mL) shows that these are non-cytotoxic.

**Figure 13:**
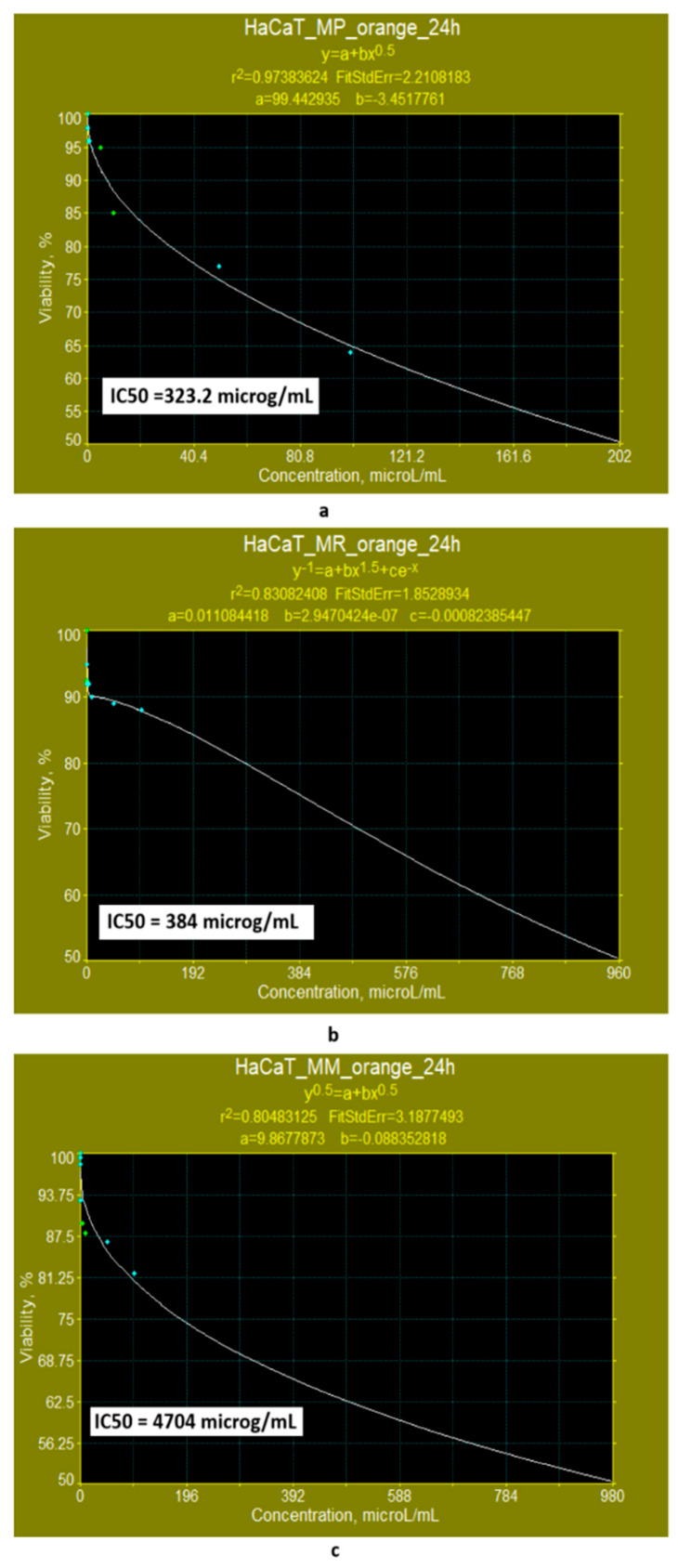
Evaluation of the IC50 value after 24 h of exposure, with nonlinear models supplied by the SYSTAT programme for orange polyketides derived from: (**a**) *Monascus purpureus*; (**b**) *Monascus ruber*; (**c**) high-productive *Monascus* sp.

The use of nonlinear models provided by the Systat programme to evaluate IC50 (Figure 13a,b), indicates that the orange polyketides derived from *Monascus purpureus* and *Monascus ruber* exhibit low cytotoxicity (IC50 = 323.2 μg/mL and IC50 = 384 μg/mL, respectively) (Figure 13a,b). In the case of orange polyketides derived from high-productive *Monascus* sp., predictions made with the Systat programme show that these are non-cytotoxic (Figure 13c), with an IC50 of 4704 μg/mL.

**Figure 14:**
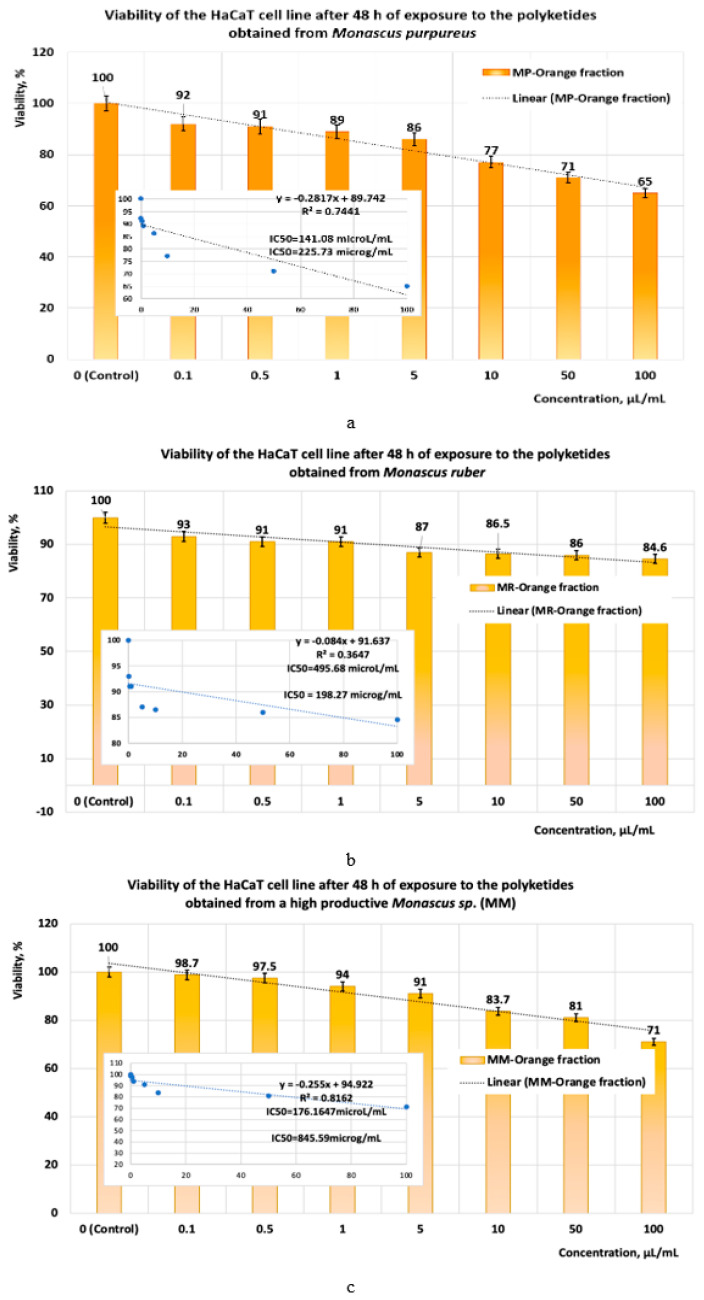
The influence of orange *Monascus*-derived polyketides on HaCaT cell line viability after an exposure time of 48 h. Evaluation of IC50 after 48 h of exposure, with linear models for orange polyketides derived from (**a**) *Monascus purpureus*; (**b**) *Monascus ruber*; (**c**) high-productive *Monascus* sp.

**Figure 15:**
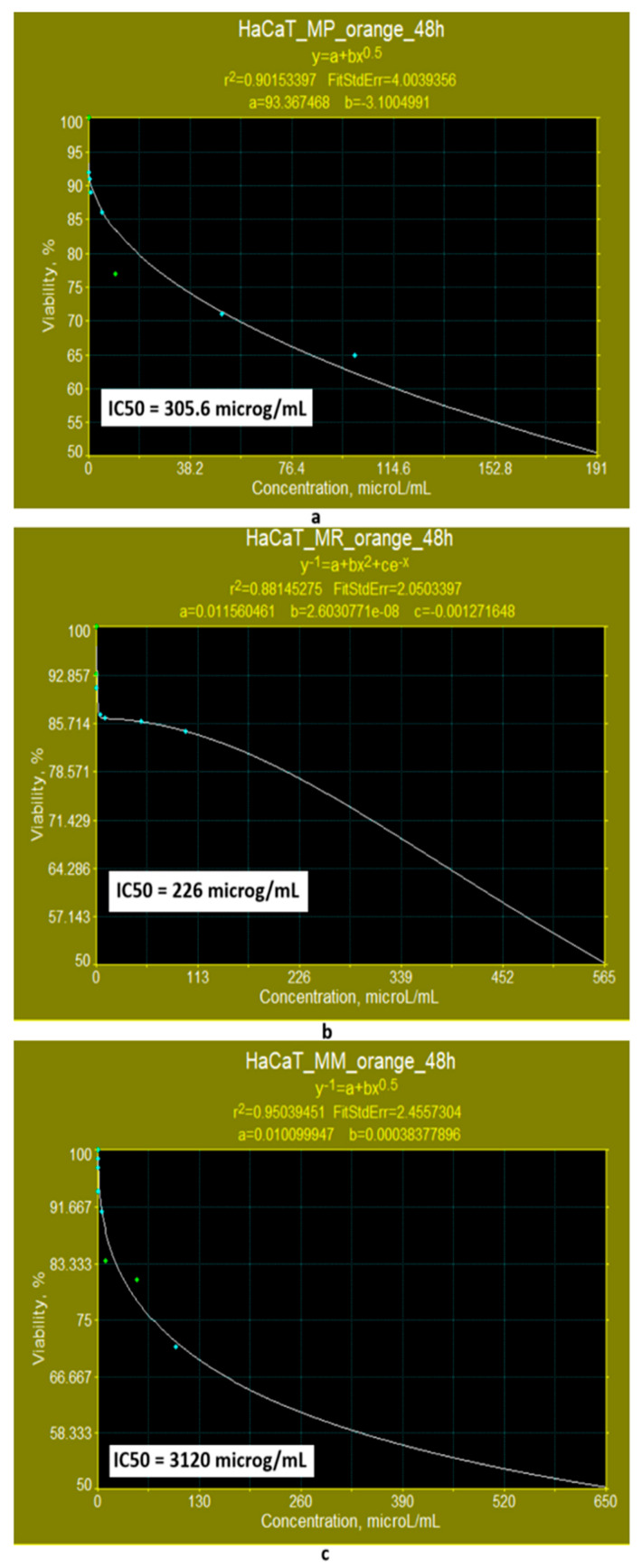
Evaluation of the IC50 value after 48 h of exposure, with nonlinear models supplied by the SYSTAT programme for orange polyketides derived from (**a**) *Monascus purpureus*; (**b**) *Monascus ruber*; (**c**) high-productive *Monascus* sp.

If the exposure time is extended to 48 h, the results obtained for IC50 with linear math models (Figure 14a–c) show that the bioproducts containing orange polyketides derived from *Monascus purpureus* and *Monascus ruber* (Figure 14a,b) exhibit low cytotoxicity (IC50 = 225.73 μg/mL and IC50 = 198.27 μg/mL, respectively). For orange polyketides derived from high-productive *Monascus* sp., the evaluation of the IC50 parameter with a linear model shows that they exhibit low cytotoxicity (IC50 = 45.59 μg/mL) (Figure 14c). Predictions made with the Systat model (Figure 15a–c) indicate that in the case the polyketides derived from *Monascus purpureus* and *Monascus ruber* (Figure 15a,b), cytotoxicity is low (IC50 = 305.6 μg/mL and IC50 = 226 μg/mL, respectively). Predicted value of IC50 for orange polyketides derived from high-productive *Monascus* sp. (Figure 15c) show that these are non-cytotoxic (IC50 = 3120 μg/mL).

**Figure 16:**
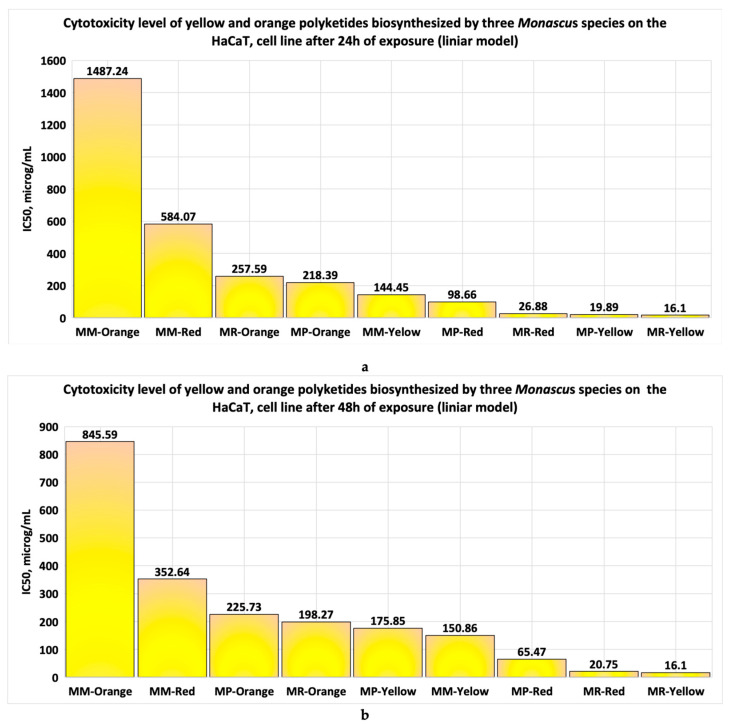
The evaluation of IC50 values of bioproducts derived from *Monascus* sp. using math linear models for (**a**) the data obtained in vitro after 24 h exposure of the HaCaT cell line for each type of *Monascus*-derived bioproduct; (**b**) the data obtained in vitro after 48 h exposure of the HaCaT cell line for each type of bioproduct tested.

The IC50 values obtained with linear models provided by Microsoft Office, after 24 h of exposure of the cell line to the studied polyketides (Figure 16a), reveal the following:

(1) The yellow polyketides derived from *Monascus ruber* and *Monascus purpureus* (Figure 16a) exhibit high cytotoxicity. In contrast, the red polyketides derived from *Monascus purpureus* and *Monascus ruber* show moderate cytotoxicity (20 μg/mL < IC50 < 100 μg/mL). The yellow and red polyketides derived from high-productivity *Monascus* sp., as well as the orange polyketides derived from *Monascus purpureus* and *Monascus ruber*, exhibit low cytotoxicity. Orange polyketides derived from high-productivity *Monascus* sp. show no cytotoxicity (Figure 16a and Table 1).
(2) The data obtained after 48 h of exposure show that the yellow polyketides derived from *Monascus ruber* (Figure 16b, Table 1) exhibit high cytotoxicity. The red polyketides derived from *Monascus purpureus* and *Monascus ruber* exhibit moderate cytotoxicity. The orange polyketides derived from *Monascus ruber* and *Monascus purpureus*, the yellow polyketides derived from *Monascus purpureus* and from high-productivity *Monascus* sp., as well as the red polyketides derived from high-productivity *Monascus* sp. (Figure 16b, Table 1) exhibit low cytotoxicity (100 μg/mL < IC50 < 1000 μg/mL). The orange polyketides derived from high-productivity *Monascus* sp. are non-cytotoxic (IC50 > 1000 μg/mL).

**Figure 17:**
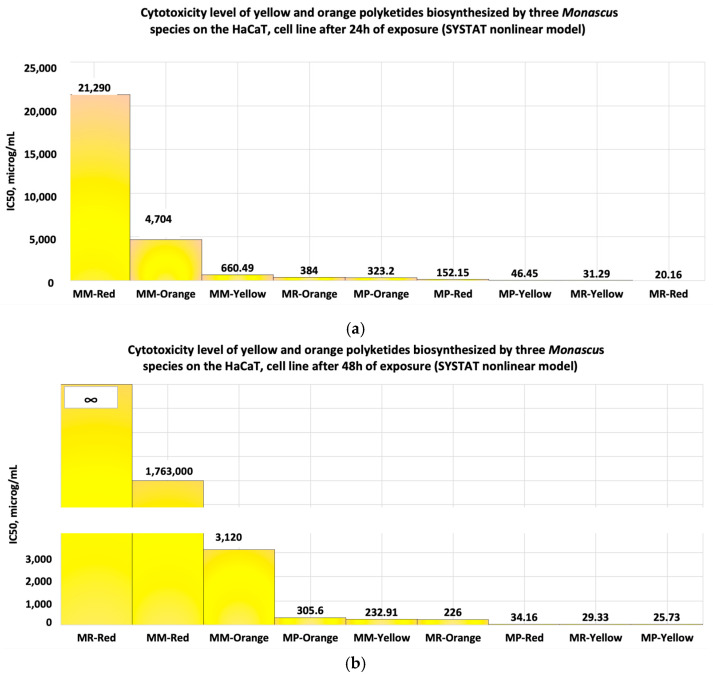
The evaluation of IC50 values for three types of bioproducts derived from *Monascus* sp. using math nonlinear models supplied by the SYSTAT programme for (**a**) the data obtained in vitro after 24 h exposure of the HaCaT cell line to each type of *Monascus-derived* bioproduct; (**b**) the data obtained in vitro after 48 h exposure of the HaCaT cell line to each type of bioproduct tested.

The IC50 values obtained with nonlinear models provided by the SYSTAT program, after 24 h of exposure of the cell line to the studied polyketides (Figure 17a), reveal the following:

1. The red polyketides derived from *Monascus ruber* and the yellow polyketides derived from *Monascus purpureus* and *Monascus ruber* (Figure 17a) show moderate cytotoxicity. The other polyketides show low cytotoxicity, except for the orange and red polyketides derived from high-productive *Monascus* sp., which are non-cytotoxic (IC50 > 1000 μg/mL);
2. The data obtained after 48 h of exposure show that the yellow polyketides derived from *Monascus purpureus* and *Monascus ruber*, as well as the red polyketides derived from *Monascus purpureus* (Figure 17b, Table 1), exhibit moderate cytotoxicity (20 μg/mL < IC50 < 100 μg/mL). The orange polyketides derived from *Monascus purpureus* and *Monascus ruber*, as well as the yellow polyketides derived from high-productive *Monascus* sp. (Figure 17b), exhibit low cytotoxicity (100 μg/mL < IC50 < 1000 μg/mL). The orange polyketides and the red polyketides derived from high-productive *Monascus* sp., and the red polyketides derived from *Monascus ruber*, are non-cytotoxic (IC50 > 1000 μg/mL).

**Figure 18:**
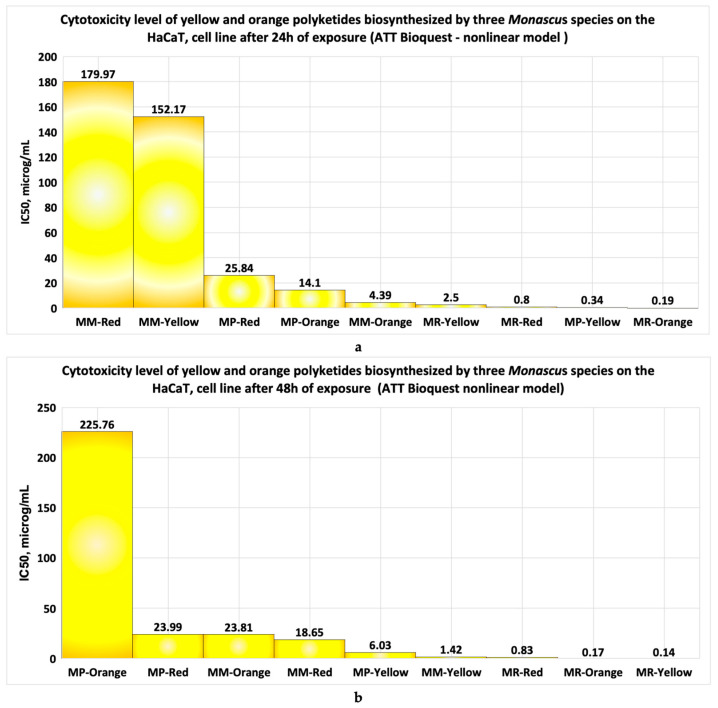
The evaluation of the IC50 value for three types of bioproducts derived from *Monascus* sp. using math nonlinear models supplied by the ATT Bioquest model for (**a**) the data obtained after 24 h exposure of the HaCaT cell line to each type of *Monascus*-derived bioproduct; (**b**) the data obtained in vitro after 48 h exposure of the HaCat cell line to each type of bioproduct tested.

The application of the ATT BioQuest model for obtaining the IC50 values after 24 h of exposure (Figure 18a and Table 1) shows that most of the studied bioproducts exhibit very high cytotoxicity (IC50 < 10 μg/mL) or high cytotoxicity (IC50 < 20 μg/mL. The red polyketides derived from *Monascus purpureus* exhibit moderate cytotoxicity (20 μg/mL < IC50 < 100 μg/mL); the red and yellow polyketides derived from high-productive *Monascus* sp. exhibit low cytotoxicity (100 μg/mL < IC50 < 1000 μg/mL) (Figure 18a). The application of the same model for the in vitro data obtained after 48 h of exposure (Figure 18b and Table 1) indicates that the orange polyketides derived from *Monascus purpureus* have low cytotoxicity (100 μg/mL < IC50 < 1000 μg/mL). The red polyketides derived from *Monascus purpureus* and the orange polyketides derived from high-productive *Monascus* species exhibit moderate cytotoxicity (20 μg/mL < IC50 < 100 μg/mL). The remaining bioproducts exhibit high cytotoxicity (red polyketides derived from high-productive *Monascus* sp.,) or very high cytotoxicity (bioproducts containing yellow polyketides derived from all *Monascus* species studied, and bioproducts containing red and orange polyketides derived from *Monascus ruber*), for which the IC50 < 10 μg/mL (Figure 18b and Table 1).

## 4. Discussions

Analysis performed in silico for six microbial polyketides biosynthesized by *Monascus* species reveals that the polyketides show pronounced lipophilicity (WlogP_o/w_ (3 ÷ 4)), making them promising materials for dermato-cosmetic formulations [13,19,20,21,22,23]. Ankaflavin, monascorubramine, and monascorubrin exhibit high molecular flexibility, which supports their antioxidant and antitumor properties [4,6,10,12,14]. Rubropunctatin, rubropunctamine, monascorubramine, and monascorubrin, due to their high degree of unsaturation, are chemically unstable, limiting their use in topical formulations due to potential degradation or adverse interactions. Various strategies, including spray drying, freeze drying, incorporation into liposomes or in the core–shell structures [5], and chemical derivatisation, can be used to enhance the stability and functionality of *Monascus* polyketides [5,37,38]. Long et al. incorporated red polyketides derived from *Monascus* sp. (highly unstable pigments) into liposomes to improve their stability and biological activity. Unilamellar, spherical liposomes with diameters ranging from 20 to 200 nm were obtained using the ultrasound-assisted thin film method. The resulting nanomaterials exhibited increased stability to variations in pH, temperature, light, and the presence of metal ions, compared to the unencapsulated red polyketides [37]. Xu and collaborators improved the stability of *Monascus* orange polyketides (also highly unstable pigments) by derivatising them with a soy protein. The resulting products were used to prepare microgels, which showed 20% greater stability to variations in temperature, pH, and light compared to the initial orange polyketides [38]. The molecules show a low capacity to pass through skin layers (logKp = (−5 ÷ −7)), monascin and rubropunctatin having negligible permeation [33,34]. The tests performed in silico by Albisoru et al. [13] demonstrated that the *Monascus*-derived polyketides exhibit toxicity when administered orally. However, these molecules display a low capacity to penetrate the skin barrier. Their use in dermato-cosmetic applications is justified by the minimal systemic absorption at the cutaneous level, which limits the body’s exposure to toxic concentrations. At the local level, on the surface of the epidermis, *Monascus*-derived polyketides may exhibit several beneficial effects, such as (a) protection of the skin against oxidative stress and ageing, due to their antioxidant and anti-ageing properties [12,13]; (b) stimulation of skin regeneration and hydration through the genic modulation of keratinocytes and fibroblasts [5,6,7,9,22,23]; (c) potential use as active ingredients in bioproducts for acne-prone skin, due to their antimicrobial activity [13]; and (d) use as active compounds in the formulation of dermato-cosmetic bioproducts for skin photoprotection [21,23]. The “boiled egg” diagrams indicate that the studied polyketides are not P-glycoprotein substrates, suggesting easy absorption from the gastrointestinal tract and the potential to cross the blood–brain barrier, except for rubropunctamine. All of these indicate that the bioproducts that contain Monascus-derived polyketides are safer if they are used in topical formulations for skincare protection [35,36]. The same considerations are valid for the spent biomass of *Monascus*, which can contain traces of these compounds, when it is used for agricultural or environmental protection applications [39,40]. A comparative analysis of the data (excluding the results obtained using ATT Bioquest), presented in Table 1, reveals that both linear and nonlinear models provided by SYSTAT software yield similar results at evaluating in vitro cytotoxicity. After 24 h of exposure, the red polyketides derived from high-productive *Monascus* sp. exhibited low cytotoxicity or were non-cytotoxic [26,28,29,30,41]. The yellow polyketides derived from high-productive *Monascus* sp. showed low cytotoxicity in both models, while the orange polyketides from the same strain were non-cytotoxic, according to data from both approaches (Table 1). Following 48 h of exposure, the linear model indicated low cytotoxicity for the red polyketides derived from high-productive *Monascus* sp., whereas the nonlinear model suggested that the bioproducts are practically non-cytotoxic. For the orange polyketides derived from the same strain, the linear model yielded an IC50 = 845.59 μg/mL, corresponding to low cytotoxicity, while the nonlinear modelling performed with SYSTAT presented IC50 = 3120 μg/mL, categorising these bioproducts as non-cytotoxic.

Regarding the yellow polyketides derived from high-productive *Monascus* sp., both math models indicated that the bioproduct exhibits low cytotoxicity. Overall, the information generated by the two mathematical approaches was significantly more consistent compared to the IC50 values obtained with ATT Bioquest software.

This discrepancy arises because the ATT BioQuest model was designed for cases where the biological effect is driven by a single compound or by a very well-defined interaction between the compounds and the biological target. In complex mixtures, where multiple compounds may contribute differently to the overall effect (through synergy, antagonism, or additivity), the BioQuest model can either underestimate or overestimate the IC50 value [42,43,44].

In the present case, the in vitro tested bioproducts contain only one class of coloured polyketides (yellow or orange or red polyketides), as well as other colourless compounds biosynthesised by *Monascus*, such as monacolines, which are found in bioproducts that contain orange or red polyketides obtained by extraction with selective solvents [45,46,47]. For bioproducts with complex compositions, flexible nonlinear models, such as those provided by MS Office or SYSTAT, with customisable settings, are more appropriate than the model supplied by ATT BioQuest for an accurate estimation of IC50 values and the assessment of cytotoxicity.

Taking into consideration the cytotoxicity limits used in the case of the tests performed in vitro [28,29,30,31,32], the use of linear models for the IC50 estimation, after 24 h of exposure, shows the following: yellow polyketides isolated from *Monascus ruber* and *Monascus purpureus* exhibit high cytotoxicity, but the red polyketides isolated from the same strains show moderate cytotoxicity. The remaining bioproducts, including polyketides derived from high-productive *Monascus* sp., exhibit low cytotoxicity, except for the orange polyketides derived from the same strain, which do not exhibit cytotoxicity.

At an exposure time of 48 h, the IC_50_ values estimated with linear models show the following: the yellow polyketides derived from *Monascus ruber* exhibit high cytotoxicity; the red polyketides derived from *Monascus ruber* and *Monascus purpureus* exhibit moderate cytotoxicity; the remaining bioproducts, including polyketides derived from highly productive *Monascus* sp., show low cytotoxicity.

The IC50 values obtained with nonlinear models provided by the SYSTAT programme, for an exposure time of 24 h, reveal that the red polyketides derived from *Monascus ruber* and yellow polyketides derived from *Monascus ruber* and *Monascus purpureus* exhibit moderate cytotoxicity; the main bioproducts, including yellow and red polyketides derived from high-productive *Monascus* sp., exhibit low cytotoxicity. The orange and red polyketides derived from high-productive *Monascus* sp. do not exhibit cytotoxicity.

After 48 h of exposure, the IC50 values obtained with SYSTAT models indicate the following: the yellow polyketides derived from *Monascus purpureus* and *Monascus ruber*, as well as red polyketides derived from *Monascus purpureus*, exhibit moderate cytotoxicity; the orange polyketides derived from *Monascus ruber* and *Monascus purpureus*, along with yellow polyketides derived from high-productive *Monascus* sp., exhibit low cytotoxicity; and the red and orange polyketides derived from high-productive *Monascus* sp. do not exhibit cytotoxicity.

The IC50 values obtained with the nonlinear model provided by the ATT BioQuest model indicate the following: after HaCat cell line exposure for 24 h to *Monascus*-derived polyketides, the main bioproducts, including orange polyketides derived from high-productive *Monascus* sp., exhibit high cytotoxicity; the red polyketides derived from *Monascus purpureus* show moderate cytotoxicity; and the yellow and red polyketides derived from high-productive *Monascus* sp. exhibit low cytotoxicity.

At an exposure time of 48 h, the IC50 values obtained using the ATT BioQuest model show the following: the main bioproducts, including red and yellow polyketides derived from high-productive *Monascus* sp., exhibit high cytotoxicity; the orange polyketides derived from high-productivity *Monascus* sp., as well the red polyketides derived from *Monascus purpureus* exhibit moderate cytotoxicity; and the orange polyketides derived from *Monascus purpureus* exhibit low cytotoxicity.

Regarding the cytotoxic properties of *Monascus*-derived polyketides, the studies on their photoprotective and antiproliferative effects are significant, particularly concerning skin health and protection issues. These effects are important for preventing tumorigenesis (melanoma) and protecting the skin from UV damage, suggesting the potential use of these compounds in skincare and melanoma therapies [23,47,48,49,50]. Wu et al. [48], in the studies performed on polyketides derived from *Monascus* sp., demonstrated their photoprotective and antiproliferative effects on a melanoma cell line. The bioproducts containing these polyketides protect skin cells from UV damage and reduce melanin production. The results suggest that these bioproducts could be an alternative for treating hyperpigmentation and sunburn [47]. Musso et al. [49], in the studies performed on natural or derivatised polyketides, showed their potential as scaffolds for creating Hsp90 inhibitors. From this point of view, the fungal polyketides offer a new approach to melanoma treatment by inhibiting Hsp90, disrupting tumour cell growth and survival. In the same view, Tan et al. [50] highlighted the ability of yellow polyketides from *Monascus* sp. to inhibit melanoma tumour cell growth, suggesting their potential for antitumor therapies.

The analysis performed in silico of six *Monascus*-derived polyketides shows that these compounds exhibit poor transdermal penetration, as indicated by low skin-permeability values, monascin and rubropunctatin displaying negligible permeation. This limited capacity to penetrate the skin barrier represents particular significance when evaluating their in vitro cytotoxicity. While certain polyketides (notably yellow and red pigments from *Monascus ruber* and *Monascus purpureus*) exhibit moderate to high cytotoxicity in vitro, depending on the exposure time and the modelling method employed, their minimal skin absorption suggests a reduced risk of systemic toxicity upon topical application. In particular, polyketides derived from high-productive *Monascus* sp., which exhibit low or negligible cytotoxicity after (24–48) hours of exposure, appear to be suitable for dermato-cosmetic applications.

The absence of P-glycoprotein-mediated efflux and the inability to cross the skin barrier reinforce the notion that these compounds exert their biological effects primarily at the epidermal level, reducing potential harm to underlying tissues or systemic circulation. The low skin permeation of the *Monascus*-*derived* polyketides serves as a protective factor, mitigating the cytotoxic potential identified in vitro and in silico, supporting the inclusion of these in topical bioproducts with antioxidant, anti-ageing, antimicrobial, and photoprotective properties.

Regarding the benefits and risks associated with *Monascus*-derived polyketides’ use in dermato-cosmetic applications at this moment, we can make the following suggestions:

(1) Polyketides biosynthesised by *Monascus* species are attracting increasing interest in dermato-cosmetic formulations, due to their wide spectrum of biological activities and natural origin. In silico and in vitro analyses reveal that these compounds, particularly red, orange, and yellow pigments, possess promising features suitable for topical formulations. However, their application must be carefully balanced with their chemical and biological limitations.
(2) From a functional point of view, *Monascus*-derived polyketides demonstrate multiple dermatological benefits. Compounds such as ankaflavin, monascorubramine, and monascorubrin exhibit high molecular flexibility, which supports potent antioxidant activity, contributing to skin protection against oxidative stress and premature ageing. These polyketides modulate genic expression in keratinocytes and fibroblasts, suggesting their potential to stimulate skin regeneration, improve hydration, and support tissue repair. Moreover, these polyketides exhibit antimicrobial properties, making them suitable candidates for formulations targeting acne-prone skin. The photoprotective and antiproliferative effects of these polyketides, especially for melanoma cell lines, highlight their capacity to protect the skin from ultraviolet radiation and reduce melanin synthesis. These features make *Monascus*-derived polyketides promising ingredients for formulations designed to address skin hyperpigmentation and UV-induced damage.
(3) A significant safety advantage of these compounds lies in their poor transdermal penetration. In silico predictions indicate very low skin permeability, with logKp values ranging from –5 to –7, and negligible permeation reported for compounds such as monascin and rubropunctatin. This limited ability to cross the skin barrier significantly reduces systemic exposure and toxicity, even for compounds that display moderate-to-high cytotoxicity in vitro.
(4) Additionally, predictions based on the “boiled egg” model suggest that these compounds are not substrates for P-glycoprotein, indicating a low likelihood of efflux-related bioaccumulation in skin cells.

Nevertheless, several limitations must be addressed to enable the safe and effective inclusion of *Monascus* polyketides in dermato-cosmetic formulations. Red and orange pigments—such as rubropunctatin, rubropunctamine, and monascorubrin—are highly unsaturated and chemically unstable, making them susceptible to degradation in the presence of light, oxygen, pH fluctuations, and metal ions. Without adequate stabilisation, these compounds pose the risks of degradation and loss of bioactivity during storage or application. Strategies such as encapsulation in liposomes, derivatisation with soy protein, followed by incorporation into microgels or in the core–shell systems have been shown to significantly enhance pigment stability and protect them from environmental stressors.

In vitro cytotoxicity data further emphasise the need for careful consideration. While red and orange polyketides derived from high-producing *Monascus* strains generally exhibit low or negligible cytotoxicity after 24–48 h of exposure (demonstrated in both linear and nonlinear models supplied by SYSTAT software), yellow polyketides from *Monascus ruber* and *Monascus purpureus* can exhibit moderate-to-high cytotoxicity, depending on the strain and exposure time. These findings underline the importance of selecting appropriate strains and using optimised formulations to ensure safety. Moreover, discrepancies between different cytotoxicity modelling methods, particularly between SYSTAT models and ATT BioQuest software, highlight the challenges of evaluating complex mixtures where synergistic or antagonistic interactions may occur. In this context, flexible nonlinear models are preferred for estimating IC50 values and assessing safety profiles in composite bioproducts.

Another consideration is the potential for skin pigmentation, as coloured polyketides may interact with the skin’s surface, potentially causing staining or influencing melanin synthesis. This risk, however, can be mitigated through using the controlled release systems that modulate polyketide concentration and exposure time.

*Monascus*-derived polyketides present a unique opportunity for innovation in dermato-cosmetic formulations due to their multifunctional bioactivities and favourable safety profile when applied topically. However, their successful application depends on advanced formulation strategies used to stabilise chemically sensitive polyketides. More in vitro and in vivo studies are warranted to fully elucidate their therapeutic potential and establish standardised protocols for their safe and effective use in skincare products.

The novelty of this study lies in the following aspects:

(1) To evaluate the IC50 of bioproducts containing different classes of coloured *Monascus* polyketides, the linear or nonlinear model provided by the SYSTAT programme is more suitable than the ATT Bioquest model, as the latter is not designed for bioproducts containing multiple bioactive molecules.
(2) The polyketides derived from a new, high-productive *Monascus* sp. exhibit low or no cytotoxicity.

A limitation of our studies is that the data were obtained only in silico or in vitro. More studies, both preclinical and clinical, are still needed to assess the safety of skincare bioproducts based on *Monascus*-derived polyketides.

The perspectives of this study can be considered from different points of view, such as (1) *Monascus* sp. remains a promising source of bioactive compounds for skin healthcare protection. Moreover, *Monascus* sp.-derived polyketides represent sustainable and low-carbon biotechnological application, in agreement with the principles of the European Green Deal. (2) Fungal polyketides can be produced via solid-state biosynthesis (the method with the greatest productive yields) or by submerged biosynthesis. After polyketide extraction, the spent biomass can be reused in environmental protection applications (e.g., biosorbents for heavy metals) [38] or can be used as raw materials for new bioproducts for soil fertilization [39,40]. (3)The cytotoxicities exhibited against tumour cell lines, as evidenced by in silico and in vivo models [51,52,53], can be exploited to develop new conditioning approaches of fungal polyketides, to enhance selectivity for tumour cells through targeted delivery systems.

## 5. Conclusions

The data obtained during the studies performed in vitro, and/or in silico, reveal more aspects regarding the toxicity of *Monascus*-derived bioproducts, which contain coloured polyketides. The six main microbial polyketides derived from *Monascus* species exhibit significant lipophilic characteristics (the corresponding WlogP_o/w_ values range between 3 and 4), making them suitable candidates for dermato-cosmetic formulations. Their affinity for the lipidic phase suggests their potential for use as active ingredients for skincare products. Ankaflavin, monascorubramine, and monascorubrin, due to their high molecular flexibility, may interact with diverse biological targets, including melanoma tumour cells, exhibiting a potential role in the antiproliferation of tumour skin cells. Their antioxidant antimicrobial and antitumor properties support their value in therapeutic and cosmetic applications. Due to their high unsaturation, the polyketides rubropunctatin, rubropunctamine, monascorubramine, and monascorubrin exhibit chemical instability, which may limit their use in topical formulations due to rapid degradation or undesired interactions with other ingredients. In silico permeability tests indicate that these polyketides possess a low capacity to cross the epidermal barrier, particularly monascin and rubropunctatin, which show negligible absorption. In vitro cytotoxicity tests on HaCaT cell lines show that yellow polyketides derived from *Monascus purpureus* and *Monascus ruber* exhibit moderate cytotoxicity, especially after 48 h exposure. The IC50 values suggest weak cytotoxicity for polyketides from the high-productive *Monascus strain*. The orange polyketides derived from *Monascus ruber* and *Monascus purpureus* exhibit low cytotoxicity, while those derived from highly productive strains do not exhibit cytotoxic effects. Both red and yellow polyketides from *Monascus purpureus* and *Monascus ruber* exhibit moderate cytotoxicity, while the orange and red polyketides derived from highly productive strains are non-cytotoxic. Cytotoxicity increases with longer exposure times (48 h), with the yellow and red polyketides maintaining or increasing their cytotoxic character, while orange polyketides from highly productive *Monascus* strains remain largely non-cytotoxic. The polyketides with moderate to low cytotoxicity, such as those derived from *Monascus purpureus* and *Monascus ruber*, and in particular the polyketides derived from high-productive *Monascus* sp., could be explored for pharmaceutical or cosmetic uses, with a focus on balancing efficacy and safety. The findings suggest that formulations using these polyketides may be safe for topical use, provided their chemical stability is considered.

## Figures and Tables

**Table 1:** Cytotoxicity level of the Monacus-derived bioproducts with red, orange, or yellow polyketides.

*Strain*	Product	IC50, μg/mL Linear Model	IC50, µg/mL Nonlinear Model	Observations	References
Exposure time: 24 h					
*Monascus purpureus*	MP-Red	98.66	152.15	Moderate cytotoxicity–low cytotoxicity	[26,28,29,30,36]
*Monascus ruber*	MR-Red	26.88	20.60	Moderate cytotoxicity for both models	
*Monascus high-productive strain*	MM-Red	584.07	21,290.00	**Low cytotoxicity–no cytotoxcity**	
*Monascus purpureus*	MP-Yellow	19.89	46.50	High cytotoxicity–moderate cytotoxicity	
*Monascus ruber*	MR-Yellow	16.10	31.29	High cytotoxicity–moderate cytotoxicity	
*Monascus high-productive strain*	MM-Yellow	144.45	660.49	**Low cytotoxicity for both models**	
*Monascus purpureus*	MP-Orange	218.39	323.20	Low cytotoxicity for both models	
*Monascus ruber*	MR-Orange	257.60	384.00	Low cytotoxicity for both models	
*Monascus high-productive strain*	MM-Orange	1487.24	4704.00	**No cytotoxicity for both models**	
**Exposure time: 48 h**					
*Monascus purpureus*	MP-Red	65.47	34.16	Moderate cytotoxicity for both models	[26,28,29,30,36]
*Monascus ruber*	MR-Red	20.75	**∞**	Moderate cytotoxicity–no cytotoxicity	
*Monascus high-productive strain*	MM-Red	352.64	1763 × 10^3^	**Low cytotoxicity–no cytotoxicity**	
*Monascus purpureus*	MP-Yellow	175.85	25.73	Low cytotoxicity–moderate cytotoxicity	
*Monascus ruber*	MR-Yellow	16.10	29.33	High cytotoxicity–moderate cytotoxicity	
*Monascus high-productive strain*	MM-Yellow	150.86	232.91	**Low cytotoxicity for both models**	
*Monascus purpureus*	MP-Orange	225.73	305.60	Low cytotoxicity for both models	
*Monascus ruber*	MR-Orange	198.27	226.00	Low cytotoxicity for both models	
*Monascus high-productive strain*	MM-Orange	845.59	3120.00	**Low cytotoxicity**–**no cytotoxicity**	

IC50 < 10 µg/mL: very high cytotoxicity; 10 µg/mL < IC50 < 20 µg/mL: high cytotoxicity; 20 µg/mL < IC50 < 100 µg/mL: moderate cytotoxicity; 100 µg/mL < IC50 < 1000 µg/mL: low cytotoxicity; IC50 > 1000 µg/mL: *no cytotoxicity*.

## Data Availability

The raw data supporting the conclusions of this article will be made available by the authors on request.
